# Fibroblast heterogeneity and its role in generating protective immunity in the secondary lymphoid organs

**DOI:** 10.3389/fimmu.2025.1519789

**Published:** 2025-04-03

**Authors:** Hadida Yasmin, Remya P. G. Ramesh, Ann Mary Joseph, Uday Kishore

**Affiliations:** ^1^ Immunology and Cell Biology Laboratory, Department of Zoology, Cooch Behar Panchanan Barma University, Cooch Behar, West Bengal, India; ^2^ Department of Veterinary Medicine (CAVM), UAE University, Al Ain, United Arab Emirates; ^3^ Zayed Centre for Health Sciences, UAE University, Al Ain, United Arab Emirates

**Keywords:** fibroblasts, lymph node, spleen, Peyer’s patches, germinal center, inflammation, extracellular matrix

## Abstract

Fibroblasts are cells of mesenchymal origin with a range of phenotypic diversity and heterogeneity. One of the major functions of fibroblasts is the formation and turnover of the extracellular matrix and establishing a tissue structure by forming a matrisome from embryonic development to the adult stage. It plays an indispensable role in extracellular matrix remodeling during injury, repair, and infection, providing a scaffold for cell-to-cell interaction. Despite their important pathophysiological roles, molecular markers for tissue-resident fibroblasts are only now being identified. Fibroblasts acquire molecular signatures based on anatomical locations, thus impacting their phenotypic heterogeneity despite their overlapping morphology. Fibroblasts are now recognized as key immune sentinel cells, capable of regulating the inflammatory milieu through their distinct functional subsets that are designed to respond differently with unique immune signatures. Fibroblasts can detect pathogenic and danger signals through their diverse pattern recognition receptors (PRRs) and release soluble mediators that can modulate the immune infiltrates at the site of tissue injury and repair. This review discusses the diversity and heterogeneity of fibroblasts in secondary lymphoid organs such as lymph nodes, spleen, and Peyer’s patches, and their contributions to a range of pathological and physiological processes. The role of trans-differentiated effector fibroblast phenotypes that modulate the expression and function of various innate immune components (PRRs, cytokines, chemokines, and complement) in maintaining homeostasis has also been discussed.

## 1 Introduction

Fibroblasts exist within most loose and dense fibrous connective tissue. After endothelial and blood cells, fibroblasts are the most widespread cell types in the human body. They reside in the visceral organ capsules, skin, gastrointestinal and urogenital tracts, airway linings, central nervous system meninges, muscle and adipose tissues, as well as the musculoskeletal system. Fibroblasts are irregular-shaped cells (elongated, polygonal, or stellate), and their cytoplasm tapers off into several processes. The nucleus is usually centrally located, occupying almost 50% of the cell volume in resting conditions. In its proliferating stage, the nucleus occupies a larger portion of the cell, and there can be more than one nucleus (two to three). Mesenchymal cells found in the developing embryo and fetus are the progenitor cells of different types of fibroblasts, which are usually surrounded by collagen fibers, being produced by them (1). Along with collagen (of which collagen I is most abundant), fibroblasts produce a range of proteins that become the base of the extracellular matrix (ECM). Vimentin intermediate filaments, fibronectin, and interstitial collagens are predominant in the ECM. During tissue injury, fibroblasts transdifferentiate into myofibroblast phenotype with contractile properties to resolve the wound; in case the wound is not resolved, it leads to fibrosis (2).

At different stages of life, starting from the fetal to adult stage, fibroblasts undergo programmed phenotypic transitions to perform a diverse range of functions—from embryonic development to the aging process. This phenotypic differentiation is tightly regulated in a tissue- and developmental stage-specific manner. The relative distribution of fibroblasts at diverse locations throughout the human body suggests its role in coordinating with the adjacent resident cells. The heterogeneity in the phenotypic variations is reflected in the cellular morphology in both external and internal milieu, expression of surface receptors, and biosynthesis and secretion of growth factors and chemoattractant molecules (3–6). Fibroblast differentiation is governed by the degree of clonal expansion, clonal deletion/diminution, or inhibition of a particular subtype. This event is regulated by the release of humoral factors by the fibroblast itself as well as neighboring epithelial and infiltrating immune cells that instruct the migration and accumulation of the different subtypes that maintain the dynamic equilibrium of the heterogeneity and diversity of the fibroblasts (7). The markers that distinguish fibroblasts from other cell types are vimentin (VIM), fibroblast-specific protein 1 (FSP1), platelet-derived growth factor receptor-alpha (PDGFRα), fibroblast activation protein-alpha (FAP), CD90 (Thy 1), podoplanin (gp38), alpha smooth muscle actin (α-SMA), and CD34 (8). There is no single specific marker to discriminate fibroblasts from other cell types in different organs due to the profound heterogeneity of fibroblasts. Muhl et al have recently interrogated, via single-cell analysis, signatures that identify and distinguish between fibroblast, vascular smooth muscle cells and pericytes of heart, colon, bladder and skeletal muscle (9). Ninety gene signatures were identified with inter- and intra-organ fibroblast heterogeneity along with cross organ similarity, mostly related to genes of matrisome and ECM. Under various pathophysiological conditions, fibroblasts undergo differentiation into effector phenotypes, some specialized for ECM production and remodeling of tissues while others for carrying out immunological functions; these include the control of ECM synthesis via secretion of varied chemokines, cytokines, and growth factors.

Secondary lymphoid organs (SLOs) play an indispensable role in compartmentalizing immune cells for an effective environment for the maintenance of host immunity and simultaneously minimizing pathological damage. Here, resident fibroblasts coordinate the immune functioning with its vast reticular conduit network. This review highlights how fibroblasts function as sentinel cells, a source of scaffold for other immune cells during normal and inflammatory conditions, being a key regulator of germinal center (GC) formation, and in the maintenance of an overall core structural organization of the SLOs, such as lymph nodes, spleen, and Peyer’s patches.

## 2 Diversity and heterogeneity of fibroblasts in secondary lymphoid organs

SLOs are intricately placed throughout the human body to perform various functions, such as receiving immunological signals from different locations, antigen sampling, and disseminating them via antigen-presenting cells (APCs). The signals received during the interaction between pattern recognition receptors (PRRs) with pathogen-associated molecular patterns (PAMPs) and damage-associated molecular patterns (DAMPs) present in pathogens, tumors, commensals, and inflamed tissues are sampled and presented to immune cells that are distributed in special niches inside the SLOs. The immune cells present in the different compartments of SLOs undergo effector functions in coordination with the lymphoid organ fibroblasts, which form the scaffold to provide the appropriate microenvironment and determine the strength of protective innate and adaptive immunity. SLOs maintain a similar organized architecture, segregated B- and T-cell compartments where lymphocyte priming and differentiation take place, and the antigen sampling zone, which harbors specialized myeloid cells. These compartments offer the microenvironments for immune cell-to-cell interaction (10).

### 2.1 Lymph node

**Figure 1:**
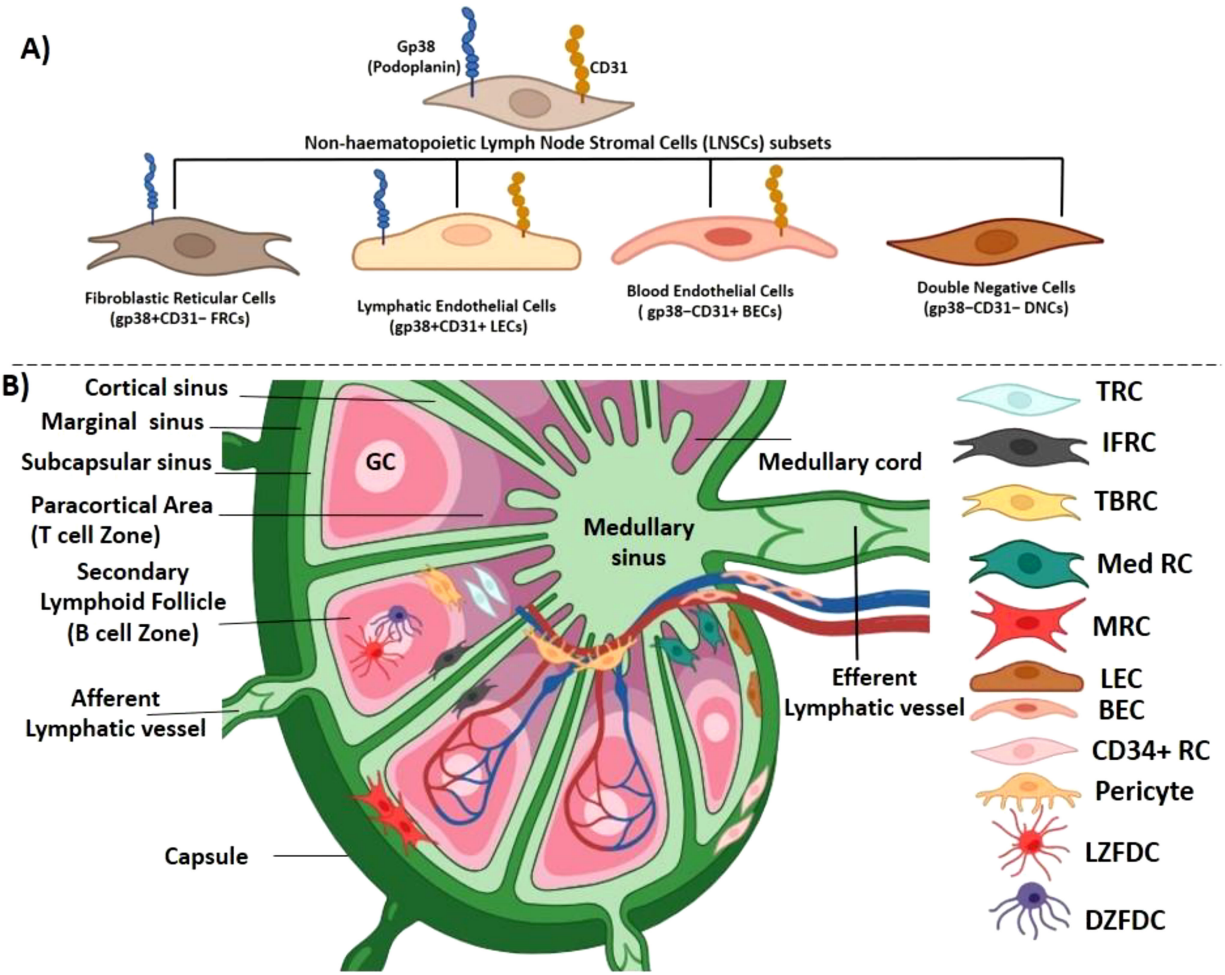
Location of different fibroblast subpopulations in lymph nodes. **(A)** The lymph node harbors an array of non-hematopoietic lymph node stromal cells (LNSCs), and on the basis of two surface markers, podoplanin (gp38) and platelet endothelial cell adhesion molecule 1 (CD31), they can further be divided into fibroblastic reticular cells (FRCs; gp38^+^CD31^−^), lymphatic endothelial cells (LECs; gp38^+^CD31^+^), blood endothelial cells (BECs; gp38^−^CD31^+^), and double-negative cells (DNCs; gp38^−^CD31**^−^**). **(B)** Lymph node is a collagenous fibrous capsulated structure, divided into cortex, paracortex and medulla. Lymph node receives lymphatic fluid through afferent lymphatic vessel, which pierces into the capsule through the subcapsular sinus (a bowl-shaped cavity) separating the capsule from the cortex. The cortical sinus originating from the marginal sinus joins into the large medullary sinuses in the medulla. This divides the lymphoid parenchyma into several small medullary cords. The medullary sinus, which is confluent with the marginal sinus, finally continues into the efferent lymphatic vessels. The immune cells follow this route to enter and exit the lymph node. The cortex consists of lymphoid follicles, which is the main B-cell zone, and the paracortical area is a T-cell zone. There are four major types of FRCs in the lymph node: T-cell zone FRCs (TRCs) distributed at the deeper T-cell zone, follicular dendritic cells (FDCs) located at the cortex in the germinal center (B-cell follicles), medullary FRCs (Med RCs) located at the medullary region, and marginal reticular cells (MRCs) positioned between subscapular sinus and the outer end of B-cell follicle. FRCs at the border of T- and B-cell zones are termed TBRCs, and cells in between the follicles are IFRCs. FDCs in the light zone are termed LZFDC, and cells in the dark zone are termed DZFDC. Two endothelial lineages are also present: lymphatic endothelial cells (LECs) and blood endothelial cells (BECs). Two other types of FRCs are pericytes and CD34^+^ reticular cells.

Lymph nodes are kidney-shaped bodies (up to 2.5 cm long) occurring along the lymphatic vessels (nearly 450 in number). It is a collagenous capsule encircling highly compartmentalized structures: cortex, paracortex and medulla. The cortex has densely packed B cells (10). In the outer cortical area, follicles have B cells and follicular dendritic cells (FDCs) that supports B cell follicles. The T cells reside in the paracortex. Primary follicles have naive B cells, but secondary follicles have GCs composed of antigen-stimulated B cells, eventually destined to become plasma cells which then moves towards the medulla. The lymph nodes have a complex network across the body for collecting drained antigens from tissues transported through lymphatic vessels. There is an enormous influx of mature lymphocytes that takes place after birth. To accommodate them, the lymph node has to expand. Both mesenchymal and endothelial cells divide and differentiate; thus, the lymph node eventually grows and matures to accommodate infiltrating immune cells, collectively known as non-hematopoietic lymph node stromal cells (LNSCs). The signature molecules expressed on LNSCs are podoplanin (gp38) and platelet endothelial cell adhesion molecule 1 (CD31). Depending on these two markers, LNSCs can be further divided into fibroblastic reticular cells (FRCs; gp38^+^CD31^−^), lymphatic endothelial cells (LECs; gp38^+^CD31^+^), blood endothelial cells (BECs/gp38^−^CD31^+^), and double-negative cells (DNCs; gp38^−^CD31^−^) (11) (**Figure 1A**). Two LNSCs have been identified in naïve peripheral lymph nodes; in B-cell follicles, CD35^+^ FDCs, and in the medulla, CD31^+^ LECs, which are also positive for a 90-kDa surface protein, 10.1.1 (12). The most abundant FRC in the T zone is gp38^+^CD31^−^CD35^−^. *In situ* fluorescence microscopy revealed two gp38^+^FRC subsets: BP-3^+^CD31^−^ in the T zone and pericytes and BP-3^+^CD31^+^ in the subcapsular sinus-lining cells in mice [benzophenone-3 (BP-3) is a surface alloantigen, which is a glycoprotein in nature, which marks adult myeloid lineages in mice] (13). LECs express Lyve-1 (lymphatic vessel endothelial hyaluronan receptor 1), VEGF-R3 (vascular endothelial growth factor receptor-3), and 10.1.1 lymphatic markers; BECs show high expression of VE-cadherin, CD34, and Tie-2 (12).

At the early stages of organogenesis of SLOs such as lymph nodes and Peyer’s patches, the existence of hematopoietic lymphoid tissue inducer (LTi) in their respective anlagen is quintessential. Mice deficient in the lymphotoxin (LT) gene could not form lymph nodes and Peyer’s patches. Both *Lta* ^−/−^ and *Ltbr* ^−/−^ mice displayed no lymph node or Peyer’s patches, suggesting the essential role of the lymphotoxin-β receptor (LTβR) to which LTα and LTβ heterodimers bind in the organogenesis of SLOs (14–18). During organogenesis, mesenchymal stromal cells, also referred to as lymphoid tissue organizer (LTo) cells, interact with LTi cells. The interaction of LTi cells with LTβR and RANKL (receptor activator of NF-kB ligand) with mesenchymal stromal cells is a crucial mechanism for the development of SLOs, which further generates a positive loop by activating more LTi cells through the expression of IL-7, CCL19, and CCL21 (18–23). LTβR association with FRCs has been shown to induce the production of B-cell activating factor (BAFF), VEGF-A, and VEGF-B by B cells (24). In addition, BAFF and IL-4 together enhance LT expression by activating B cells, promoting B cell–FRC engagement. This feedback loop is thus required for mesenteric lymph node lymphangiogenesis.

The mature lymph node consists of B cells in the cortex, T cells and dendritic cells (DCs) in the paracortex, and macrophages and plasma cells in the medulla. FRCs of mesenchymal origin facilitate the migration of the infiltrating immune cells in distinct niches in the lymph node by constructing a reticular network of specialized fibers that are used as a scaffold as well as a cargo for cell-to-cell interaction. An intricate conduit system of microchannel is formed by this reticular network, facilitating the transport of chemokines and small molecules (10). FRCs in the lymph node have four broad subtypes: i) T-cell zone FRCs (TRCs), which are present at the paracortical and inter-follicular regions; ii) FDCs, which reside in the cortex supporting B-cell follicles; iii) marginal reticular cells (MRCs) located between the outer region of B-cell follicle and subcapsular sinus; and iv) medullary FRCs (Med RCs). In the adult lymph node, this heterogeneity of FRCs is essential for the homing, migration, and survival of the immune cells. Podoplanin (gp38), a mucin-like membrane glycoprotein expressed on FRCs, maintains the stiffness of the lymph node and the reticular network tension by regulating the actomyosin contractility. The physical elasticity in SLOs is also maintained by podoplanin signaling in FRCs and its modulation by CLEC-2 on DCs (25). Podoplanin can induce actomyosin contractility in FRCs via RhoA/C activation in mice. This is achieved by CLEC-2-mediated clustering of podoplanin, freeing it up from RhoA/C. Single-cell RNA-seq analysis revealed nine different subtypes of FRCs in mice and humans: TRCs, MRCs, Med RCs (Inmt^+^ and Nr4a1^+^), TBRCs (located at the border of T- and B-cell zone), FDCs, IFRCs (FRCs located in between follicles), CD34^+^ reticular cells, and pericytes (26, 27) (**Figure 1B**, **Table 1**).

**Table 1:** Fibroblast types and subtypes in SLOs based on single cell transcriptomic analysis (Ref 26–30)

Cell type	Cell Markers	Immune functions
**Fibroblast** [No single specific marker to discriminate fibroblasts from other cell types in different organs ]	Vimentin (VIM) Fibroblast specific protein 1 (FSP1) Platelet derived growth factor receptor-alpha (PDGFRα) Fibroblast activation protein-alpha (FAP) CD90 (Thy 1) Podoplanin (PDPN)/gp38 Alpha smooth muscle actin (α- SMA) CD34	An important sentinel cell A source of scaffold for the immune cells in tissues Facilitates immune coordination and functioning ( a cargo for cell to cell interaction) in tissues An important regulator in maintaining the core structure and organization of SLOs. A key regulator of germinal centre formation. An important modulator of innate immune response.
Fibroblast types and subtypes based on their location in SLOs		
Lymph node		
Cell types	Function	
FRC (Fibroblast Reticular Cells)	Creates an intricate conduit system of microchannels formed by its reticular network to support immune cell interactions. In adult lymph node, heterogeneity of FRCs is essential for the homing, migration and survival of the immune cells. Maintains the stiffness and the reticular network tension of the lymph node. Guide T cell and dendritic cell migration. Acts as a surveillance mechanism in controlling activation of self-reactive T cells. An important source of BAFF and VEGF	
Subtypes of FRC	Location and markers	Functions
A) TRC (T cell Zone FRC)	Paracortical and inter-follicular regions (*Pdpn^+^, Cd31^−^, Ccl21^+^, Ccl19^+^, Il7^+^*)	Exhibits high expression of PDGF-Rα, PDGF-Rβ, VCAM-1, ICAM-1, BP-3, TNFR, LTβ-R and TNF-R1 Secretes CCL19 and CCL21 that recruit CCR7 expressing DCs and naïve T cells at the paracortex region. Major source of IL-7
**B) MRCs** (Marginal Reticular Cells)	Between the outer region of the B cell follicle and subcapsular sinus (*Pdpn^+^, Madcam1^+^, Tnfsf11^+^, Cxcl13^+^, Vcam1^+^, BST1^+^*)	Differentiation into mature FDCs Influences antigen interaction with lymphocytes
**C) FDCs** (Follicular Dendritic Cells)	Cortex region *Pdpn^+^, Cd31^−^, Cxcl13^+^, Cd21^+^, Baff^+^, Cr2^+^*	Capturing and retaining antigens as well as immune complexes for providing them to B cells for their activation. Increases GC area to support expansion of B cell. Facilitates the production of high-affinity antibodies.
**D) Med RCs** (Medullary FRCs)	Medulla *Pdpn^+^, Cd31^−^*	Express BAFF and IL-6 to support plasma cell survival. Regulates plasma cell positioning.
**E) TBRCs** (T-B cell zone FRCs)	At the border of T and B cell zone *Pdpn^+^, Cd31^−^, Grem1^+^, Ccl19^lo^*	Absence of TBRCs led to complete loss of residential dendritic cells affecting the function of T cells.
**F) IFRCs** (Interfollicular FRC)	Reticular cells located in between follicles *Pdpn^+^, Cd31^−^, Ccl21^+^, Ccl19lo, Il7^+^, Cxcl9^+^*	Capable of expressing MHC-II-related genes Possibly helps in inducing Cd4+ T cell tolerance
**G) CD34+ reticular cells**	Found in capsule and perivascular region in medulla *Pdpn^+^, Cd31^−^, Bst^−^, Acta2^−^, Cd34^+^*	Differentiate into FRC, MRC, and FDC and pericytes
**H) Pericytes**	Around high endothelial venules (HEVs) and surrounding capillaries *Pdpn^−^, Cd31^−^, Itga7^+^, Acta2^+^*	Maintaing endothelial integrity Helps in lymphocyte recruitment to the SLOs by releasing chemotactic factors
Spleen		
Subtypes of FRC	Location and markers	Functions
**A) White Pulp FCs**	Localised in White Pulp *Bst1^+^, desmin^+^, Mfge8^+^, Clu^+^, Enpp2^+^, Il33^+^, Cxcl9^+^*	Support white pulp structure, produce extracellular matrix, regulate immune cell interactions A crucial immunomodulator regulating T reg Type-1 and Type-2 response. Regulator of T cell differentiation and migration
Subtypes of White Pulp FCs	Markers	
1. TRCs (T-zone Reticular Cells)	*Bst1^+^|Ccl21a^+^ *and* Ccl19*	
2. FDCs (Follicular Dendritic Cells)	*Bst1^+^|Cr2^+^ Cxcl13 *and *Madcam1*	
3. MRDs (Marginal reticular Cells)	*Bst1^+^|Ch25h^+^ Madcam1 *and* Tnfsf11*	
4. TRC (T cell zone FRC)	*Bst1^+^|Tnfsf13b^+^ Dpt^+^ Cxcl12^hi^, Tnfsf13b^hi^*	
**B) Red pulp FCs**	Specifically situated in the red pulp and in the marginal zone *Ly6c^+^ , Wt1, Tcf21, Csf1^hi^, Cxcl12^hi^, Coch, Cd36, Hs6st2, Npnt, Cadm4, desmin^+^*	Maintains structural integrity and organization of the spleen. Formation and maintenance of blood vessels and regulate blood filtration. Anti-viral response showing elevated IFN-activated gene expression.
Subtypes of Red Pulp and the Marginal Zone FCs	Markers	
1) Presence of heme oxygenase 1	*Ly6c1^+^|Hmox1^+^C1q^+^* *Ly6c1^+^|Hmox1^+^C1q^−^*	
2) Absence of Heme oxygenase 1	*Ly6c1^+^|Hmox1^-^Ccl5^lo^* *Ly6c1^+^|Hmox1^-^Ccl5^hi^*	
**C) FC of perivascular region** (Pericytes / Vascular smooth muscle cells)	Associated with blood vessels *Mcam^+^, Rgs5, Notch3, Cspg4, Esam, Acta2, Myh11*	Regulating blood flow. Supports nerves running along the splenic blood vessels.
**Subtypes of FCs of perivascular niche**	**Markers**	
1. Associated with both red and white pulp blood vessels	*Mcam^+^|Myh11hi FC*	
2. Associated only with specific red pulp vessels	*Mcam^+^|Cxcl13^+^ FC*	
**D) Adventitial cell** (*Cd34*+ FC)	Surrounds larger vessels, nearby the trabeculae *Pdgfra,Igfbp6, Penk, Lum, Ptgis*	Act as a progenitor of different stromal cell types.
Peyer’s Patches		
Subtypes of FRC	Location and markers	Functions
**A) MRCs** (Marginal Reticular Cells)	Subepithelial Dome *Cxcl13, Madcam1, Coch^hi^, Sncg^hi^, Sox9^hi^*	Supports antigen sampling, produce chemokines for immune cell recruitment.
**B) FDCs** (Follicular Dendritic Cells)	GC of the follicular and interfollicular area *Coch, Sncg, Sox9, Cr1/Cr2^hi^*	Supports B cell follicle formation, produce chemokines for B cell migration. Role in formation and maintenance of germinal centres. FDCs expresses CR1/CR2 with high affinity for IgM and IgA.
**C) TBRCs** (T-B cell zone FRCs)	Located in between the borders of B and T cell zones *Cxcl13, Ccl19, Ccl21a, Acta2^hi^, Madcam1^hi^*	They produce chemokines like CCL19 and CCL21, which attract T cells and dendritic cells to the lymphoid tissue. Can regulate the activity of innate lymphoid cells through IL-15 secretion.
**D) TRCs** (T-zone Reticular Cells)	Supports migration and survival of T cells by producing CCL19 and CCL21	
Subtypes of TRCs	Location and markers	
1. TRC1	At perivascular region of the T cell zone *cd34, Ly6a, Klf4, Col1a1^hi^, Col3a1^hi^, Col5a1^hi^, Col14a1^hi^, Acta2, Pdgfrb*	
2. TRC2	T cell zone *Col1a1^hi^, Lum^hi^, Ccl19, Ccl21a, Acta^lo^* (High expression of Interferon-stimulated genes)	
3. TRC3	T cell zone *Col1a1, Lum^hi^, Ccl19, Ccl21a, Acta^lo^*	

In the conduit system of lymph nodes in mice, TRCs exhibit high expression of platelet-derived growth factor receptors (PDGFRα and PDGFRβ), VCAM-1, ICAM-1, and BP-3. TRCs also show high expression of tumor necrosis factor receptors, (TNFRs), LTβ-R, and tumor necrosis factor receptor-1 (TNF-R1), and are the main source of CCL19, CCL21, and IL-7 in SLOs. In naïve lymph node, gp38^+^ TRCs co-express desmin and α-SMA, a feature of the myofibroblasts (12). FRCs are also the source of BAFF, which is essential for B-cell proliferation and maturation in the lymph node (31). These BAFF^+^ FRCs are not found in the T-cell zone. Mice with the selective depletion of FRCs, when challenged with UV-inactivated influenza A virus (IAV), showed a reduction in the B-cell number as well as in IAV-specific IgM and IgG2b antibodies. The study also revealed that the migration of B cells from high endothelial venules (HEVs) to BAFF-rich follicles was coordinated by FRCs (31). FRCs are the main VEGF-expressing cells in the T zone and the medullary cords of lymph nodes responsible for the proliferation of peripheral node addressin endothelial cells (PNAd^pos^ and PNAd^neg^) (32).

**Figure 2:**
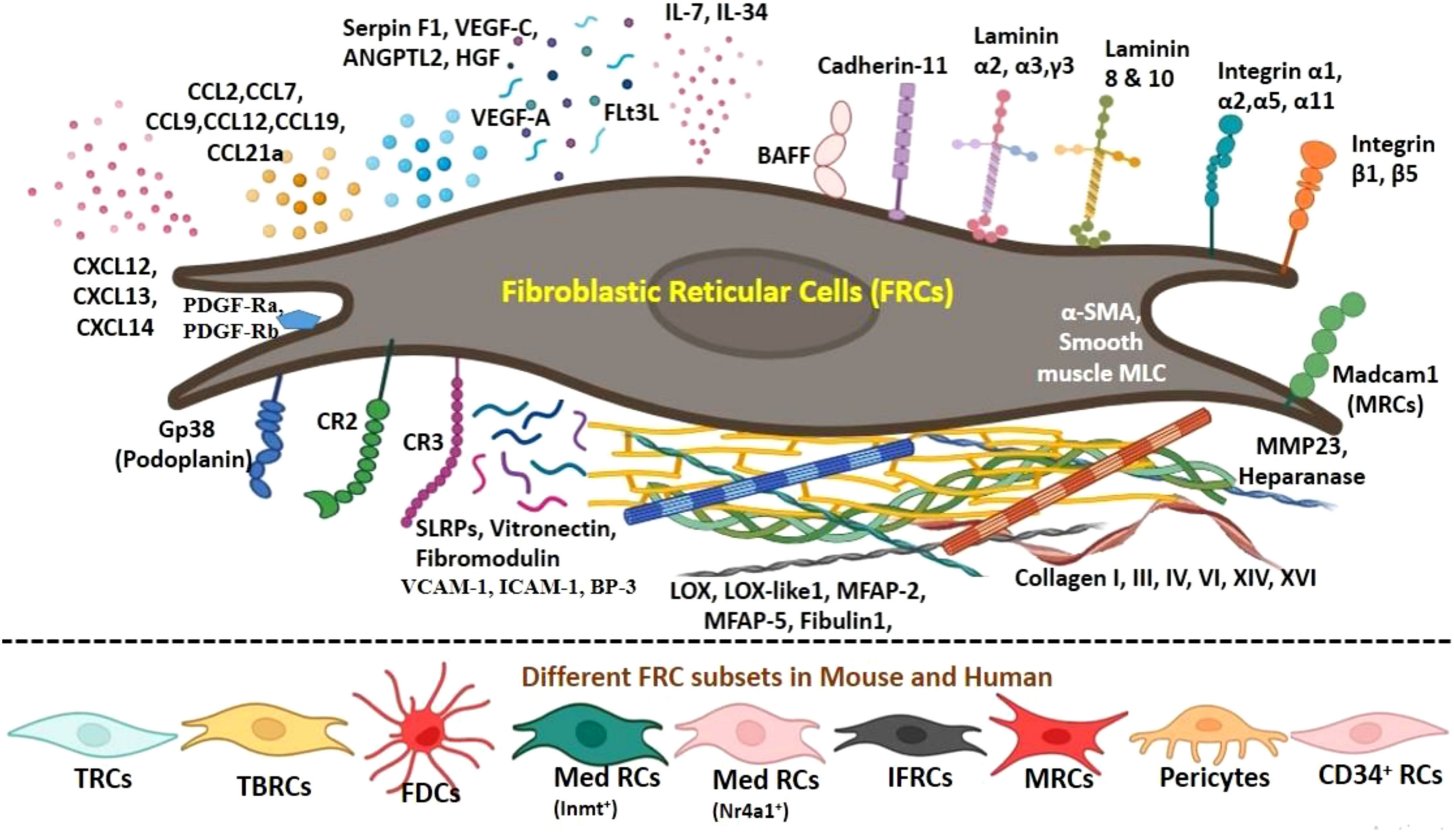
Immune signatures expressed by fibroblastic reticular cells (FRCs) and their subsets. FRCs of mesenchymal origin facilitate the migration of the infiltrating immune cells in distinct niches in the lymph node by constructing a reticular network of specialized fibers that are used as a scaffold as well as a cargo for interaction. An intricate conduit system of microchannel is formed by this reticular network facilitating the transport of chemokines and small molecules. Two important groups of chemokines being produced by FRCs are CCL and CXCL. CCL2 and CCL7 help in facilitating recruitment of T cells and dendritic cells (DCs). CCL-12, CCL-13, and CCL-19 are lymphocyte-guiding chemokines. CXCL-12 promotes homing of B cells, CXCL-13 is expressed by FDCs to attract B cells for antigen presentation, and CXCL-14 is a chemoattractant for DCs and monocytes. TRC subtypes are the main source of CCL19, CCL21, and IL-7 in secondary lymphoid organs. TRCs also show high expression of platelet-derived growth factor receptors (PDGFRα and PDGFRβ), VCAM-1, ICAM-1, and BP-3. IL-34 binds to FRCs and is the main source of B-cell activating factor (BAFF) in lymph node stromal cells (LNSCs). Collagen (I, III, IV, VI, XIV, and XVI) and integrins (α1, α2, α5, α11, β1, and β1) are synthesized by FRCs, which contributes to the remodeling of extracellular matrix. Laminin 8 and laminin 10 are expressed by each LNSC. Laminin chains α1, α2, γ3, vitronectin, and fibromodulin support the conduit structure and function. Lysyl oxidase (LOX), LOX-like protein 1 (LOXL1), fibulin 1, and microfibril-associated protein (MFAP-2 and MFAP-5) are the compounds that contribute to elastic fiber assembly and matrix formation, which are expressed by FRCs. Angiogenic regulatory molecules such as angiopoietin-related protein-2 (ANGPTL-2), VEGF-A, VEGF-C, hepatocyte growth factor (HGF), and pigment epithelium-derived factor (Serpin F1) are expressed by different FRCs. Small leucine-rich proteoglycans (SLRPs) such as lumican, osteoglycin, fibromodulin, biglycan, decorin, and propargin are also expressed.

LECs express gp38, which helps in transporting DCs from lymphatics to the lymph node parenchyma, and secrete CCL21, which directs the mobilization of the CCR7-expressing DCs toward the paracortex. TRCs secrete CCL19 and CCL21 that recruit CCR7-expressing DCs and naïve T cells to the paracortex region. BECs facilitate the entry of naïve T cells from the HEVs to the lymph node. LECs in the subcapsular sinus are of two types depending on their location: i) ceiling LECs (cLECs), which can scavenge CCL19, CCL21, and CCL25, and ii) floor LECs (fLECs), which express TNFRSF9, CCL21, and CCL25 (27). The CCL21 chemokine gradient is dependent on atypical chemokine receptor (ACKR), also known as CCRL1, which is also expressed by these LECs that are located at the ceiling of the subcapsular sinus (33). Ceiling LECs express ACKR4 (an internalizing receptor), which acts as a receptor for CCL19 and CCL21 that scavenge/degrade chemokines from the sinus lumen in order to establish a chemokine gradient to help DC migration (34). Floor LECs attract mature B cells, T-cell subtypes, DCs, and CCR6-expressing leukocytes (27). Pericytes ensheath capillaries in the lymph node to form tight junctions with HEVs and endothelial cells to maintain vascular integrity. These cells regulate smooth muscle contractility in the perivascular region by expressing α-SMA, integrin alpha 7 (Itgα7), and calponin-1 (8). **Figure 2** illustrates various signature molecules expressed by different FRCs while maintaining an appropriate microenvironment conducive to efficient innate and adaptive immune responses in the SLOs.

To induce peripheral tolerance, the APCs need to migrate to the lymph node. All LNSCs express and present an array of peripheral tissue-restricted antigens (PTAs). FRCs and LECs present a range of self-antigens; if autoreactive self-antigens are recognized by naïve CD8^+^ T cells, they are deleted by these cells (35). Tolerance is also enforced via immune suppression during normal and inflammatory conditions, which restricts overstimulation to maintain homeostasis. Prostaglandin E_2_ (PGE_2_) is a T-cell immunosuppressor, and its activity in FRC is an important mechanism of T-cell tolerance in lymph nodes. During homeostasis, the COX-2/PGE_2_ pathway in FRCs is hyperactivated to downregulate TCR signaling, and the iNOS/NO pathway is active only when T cells are fully activated. In doing so, FRCs inhibit IFN-γ and IL-2 in activated CD4^+^/CD8^+^ T cells non-antigenically and independent of the iNOS/NO pathway, thus avoiding any accidental stimulation of T cells (36). When T cells were co-cultured with FRC (*ex vivo* for 12 hours), Ca^2+^ flux was reduced. Similarly, when FRCs were removed, Zap70 phosphorylation was enhanced, suggesting FRC-mediated suppression of T-cell activation (36). FRCs in both mice and humans show suppression of T-cell activation via the COX-2/PGE_2_ pathway during homeostasis, acting as a surveillance mechanism to control the activation of self-reactive T cells.

### 2.2 Spleen

The spleen, the largest SLO, plays a central role in regulating the adaptive immune response. Similar to lymph nodes, splenic white pulp harbors T and B lymphocytes contained within distinct compartments: T lymphocytes in the periarteriolar lymphoid sheath and B lymphocytes in the follicles (37). White pulp, the lymphocyte-rich area, supports the antigen-specific immunity. The red pulp area containing macrophages and erythrocytes is an immune surveillance zone for blood-borne pathogens (10). However, the generation of antigen-specific immune responses depends largely on stroma-derived chemokines and their cellular organization. In between the white and red pulp is the marginal zone. In addition to immune cells, there are red pulp fibroblasts, endothelial cells, and mural cells (vascular smooth muscle cells and pericytes) distributed across different splenic compartments (24).

Using the Cre/LoxP system (a site-specific mammalian gene editing technology), Castagnaro et al. worked out the genetic lineage and found that all non-endothelial stromal cells originated from a mesenchymal precursor, which co-expressed NKx2-5 and Islet1. It was also found that all stromal subsets in the spleen (FRCs, MRCs, FDCs, and mural cells) descended from the NKx2-5^+^ Islet1^+^ lineage. The embryonic NKx2-5^+^ Islet1^+^ lineage defined the mesenchymal lymphoid tissue organizer (mLTo) cells, which expressed LTβR, VCAM-1, ICAM-1, CXCL-13, CXCL-19, and IL-7 (38). The non-endothelial stromal cells in the white pulp are CCL-21-expressing TRCs, CXCL-13 and CR1/CR2-expressing FDCs, and CXCL-13 MAdCAM1 and RANKL MRCs. TRCs are gp38^+^CD31^−^ ERTR-7^+^ desmin^+^, and FDCs located at the B-cell follicle are gp38^−^CD31^−^ (39). Similar to lymph nodes, TRCs, FDCs, and MRCs all express CD157; TRCs also express α-SMA, while perivascular reticular cells (PRC) express stem cell antigen-1 (Sca-1). Sca-1^+^ cells co-express PDGFRα. Two different scRNA-seq analyses were carried out to interrogate the heterogeneity of splenic FRCs; 80% of the white the pulp fibroblasts were ICAM-1^+^CD157^+^ Endoglin^−^ (28). Fibroblasts in the red pulp zone were highly Endoglin^+^, fibroblasts of the subscapsular region were lumicant^+^, and the splenic capsular outer layer fibroblasts were mesothelin^+^ (28). An additional subtype was designated as intermediate PRC (int-PRC), which expressed PRC markers (*Ly6a/Sca-1*) but lacked differentiation markers for TRC/MRC/FDC such as *Bst1*, *Enpp2*, and *Clu*. There were two clusters that were grouped as triple-negative cells (TNCs): one group expressed high mural cell markers (TNC1) such as *Pdgfrb*, *Mcam* (CD146), *Cnn1*, and *Cspg4*, and the other group expressed *Cdk6*, *Cks2*, *Mki67*, and *Mcm7*, which are cell cycle markers (TNC2). Another group of CD29^+^Calponin-1^+^ TNC1 mural cells was found around the arterioles of the white pulp. While exploring the progeny of embryonic mLTo cells, it was confirmed that the differentiation of mLTo precursors into CD21/35^+^ FDC, PDPN^+^ TRC, and MAdCAM-1^+^ MRC occurred because of *Ltbr* proficiency (28). LTβR, which is a TNF receptor superfamily member, is crucial for developing the architecture of the lymphoid tissue as well as regulating immune response against intracellular pathogens (40). The activation of mLTo cells through LTβR signaling drives the differentiation of FRC subtypes in the white pulp. Thus, the expression of LTβR in reticular subtypes is essential for the establishment of the splenic white pulp (28). However, *Ltbr* ablation did not affect the proliferation of the PRC subtypes, suggesting that they were independent of LTβR signaling.

Pezoldt et al. conducted a detailed single-cell RNA sequencing with over 20,000 splenic fibroblast cells (FCs) and classified them into four subtypes, which they distributed into four identities: a) *Bst1* ^+^ white pulp FC, b) *Ly6c1* ^+^ red pulp FC, c) *Mcam* ^+^ pericytes, and d) *CD34* ^+^ adventitial cells. *Bst1* ^+^ white pulp expressed several genes involved in lymphocyte proliferation, differentiation, and migration such as *Mfge8*, *Clu*, *Enpp2*, and type 1 modulators such as *Il-33* and *Cxcl9*. Four *Bst1* ^+^ white pulp FC clusters were identified: a) *Bst1+|Ccl21a+* was the TRC, which expressed *Ccl21* and *Ccl19*; b) *Bst1+|Cr2+* was the FDC expressing *Cr2*, *Cxcl13*, and *Madcam1*; c) *Bst1+|Ch25h+* was the MRC expressing *Ch25h*, *Madcam1*, and *Tnfsf11*; and d) *Bst1+|Tnfsf13b+* FC, located in the T-cell zone, showed upregulated expression of dermatopontin (*Dpt*) and MHC class II and were the major source of *Tnfsf13b* in the spleen. *Mcam* ^+^ FC showed markers of pericytes [*Mcam*, *Rgs5*, *Notch3*, *Cspg4* (NG2), and *Esam*], vascular smooth muscle cell (VSMC) contractility markers [*Acta2* (ASMA), *Myh11*, *Myl9*, *Lmod1*, *Cald1*, and *Tagln* (SM22)], and myocyte lineage marker *Mef2c*. *Mcam* ^+^ FC was the main source of *Ngf* and *Ntf3* (neurotrophic factors) and was divided into two subsets: *Mcam+*/*Myh11hi* (*Notch3* ^+^ *Cxcl13* ^−^ cells) found in both red and white pulp and *Mcam+|Cxcl13+* found in the red pulp around some selective blood vessels. CD34^+^ FC expressed PDGFRα, *Igfbp6*, *Penk*, *Lum*, and *Ptgis* and were located around large vessels surrounding *Mcam* ^+^ FC and found similar to *Cd34* ^+^ *Ly6c1* ^+^ *Dpt* ^+^ pan-tissue adventitial cells. The last subtype, *Ly6c1* ^+^ FC, located in the red pulp and the marginal zone expressed *Wt1* and *Tcf21* genes and the highest level of *Csf1* and *Cxcl12* compared to all splenic FCs. *Ly6c1* ^+^ FC also expressed several ECM components such as *Hs6st2* (heparan sulfate editing enzyme), *Npnt* and *Cadm4*, *Cd36* (class B scavenger receptor), *Coch* (cochlin), and desmin. These cells had two other subsets depending on the presence and absence of heme oxygenase 1 (*Hmox1*) transcript: *Ly6c1+/Hmox1+C1q+* (upregulated C1q expression) and *Ly6c1+/Hmox1+C1q−* (expression of *Gdf15*, *Nr4a1*, *Nfkbia*, *Fosb*, and *Junb*), *Ly6c1+/Hmox1−Ccl5lo*, and *Ly6c1+/Hmox1−Ccl5* ^hi^ (29).

### 2.3 Peyer’s patch

Peyer’s patches (PPs) are gut-associated lymphoid tissues, which function as intestinal immune sensors. There are approximately 180 to 240 PPs in adult humans. These intestinal follicular aggregates are surrounded by epithelium of specialized cells, known as M cells (microfold), which are capable of transporting luminal antigens. PPs are separated into three regions: a follicle-associated epithelium, a follicular area, and an interfollicular area. The GC of the follicular and interfollicular area contains B lymphocytes, macrophages, and an extensive network of FDCs. A subepithelial dome (SED) surrounding the GC contains T and B lymphocytes, macrophages, DCs, and RANKL^+^ MRCs (41, 42). The epithelium of PP is absorptive in nature, favoring fluid transport; its subepithelial location provides direct contact with intestinal pathogens (42). Immune cells carrying the antigens from PPs travel to the mesenteric lymph node. The conduit system in PPs functions similar to the lymph node where it draws intestinal fluid from the lumen supported by a reticular network of FRCs that express perlecan and podoplanin (gp38) and are CR1/CR2^+^.

PP development follows three definite steps: a) mesenchymal cells express VCAM-1 and ICAM-1, forming organizing centers; b) the accumulation of IL-7Rα^+^, CD4^+^, and Ia^+^ (immune-associated) cells; and c) the entry of CD3^+^ and B220^+^ mature lymphocytes (43). The cell aggregates arising at the early anlagen are CD3^−^CD4^+^ LTi cells. As the PP gradually matures, the FRCs differentiate into their subtypes to perform their specialized functions. Prados et al. showed at least six major FRC subtypes that are common to FRCs of lymph nodes and the spleen. The SED, which is the antigen sampling zone, contains MRCs that express *Cxcl13* and *Madcam1*, as well as FDC genes such as *Coch*, *Sncg*, and *Sox9*. FDCs in the PP express CR1/CR2 with high affinity for IgM and IgA. Three TRC subtypes (TRC1/2/3) are located at different zones in the PPs (30). TRC1 is found surrounding the blood vessels and express perivascular markers *cd34*, *Ly6a*, *Klf4*, and ECM-associated genes *Col1a1*, *Col3a1*, *Col5a1*, and *Col14a1*. TRC2 and TRC3 show high expression of *Col1a1* and *Lum* along with other TRC markers. Genes such as *Ccl19* and *Ccl21a*, which are LTβR-dependent FRC markers, are high in TRC2, TRC3, and TBRC. TBRC clusters exhibit high expression of *Acta2*, *Cxcl13*, and *Madcam1* (30). An IL-15-dependent niche is created by FRCs for the functioning of innate lymphoid cells-1 (ILC1), which are known for clearing intracellular pathogens by secreting IFN-γ. The antiviral immune responses by ILC1 and NK cells are regulated by FRCs by limiting IL-15 in PPs as well as in mesenteric lymph nodes (44). IFN-induced genes are highly expressed on TRC2. The gut has a number of SLOs that contribute to homeostasis following microbial colonization and food-derived antigen challenge. Like PPs in the small intestine, colonic patches (CPs) are composed of B-cell follicles and distinct T-cell zones, containing CD35^+^ FDCs. The existence of CP is reliant on the LTβR pathway together with CXCL13 expression (45). However, they are dependent on CCR6–CCL20 engagement compared to PPs. Curiously, CP follicles increase in numbers in IL-25-deficient mice as a result of the overexpression of IL-23 (46). As opposed to other SLOs, intestinal PP and CP formation is started after birth following exposure to food and microbial colonization (47). Thus, understanding the SLO formation of PPs and CPs may have therapeutic benefits in gut-associated inflammatory diseases given their involvement in the Th17–Th23 axis.

## 3 Role of fibroblast in the formation of germinal centers

GCs are specialized microstructures that are formed in SLOs during infection, or when challenged with T cell-dependent antigens (48). GC provides a dynamic microenvironment favoring the proliferation and differentiation of antigen-activated B cells, leading to the generation of high-affinity plasma cells and memory B cells. Mitotically active B cells undergo affinity maturation at the GCs, which are known as centroblasts/plasmablasts. The immunoglobulin modification at the variable genes of B cells takes place through somatic hypermutation to increase its affinity toward the antigen (49, 50). The emergence of plasmablasts at the interface of the germinal follicle is governed by IL-21 produced by T follicular helper (T_FH_) cells and TNFSF13 produced by gp38^+^CD157^high^ FRC. Here, the plasmablasts express TNF receptor superfamily member 13B (TNFRSF13B), which can bind TNFSF13, enhancing its proliferation (51).

**Figure 3:**
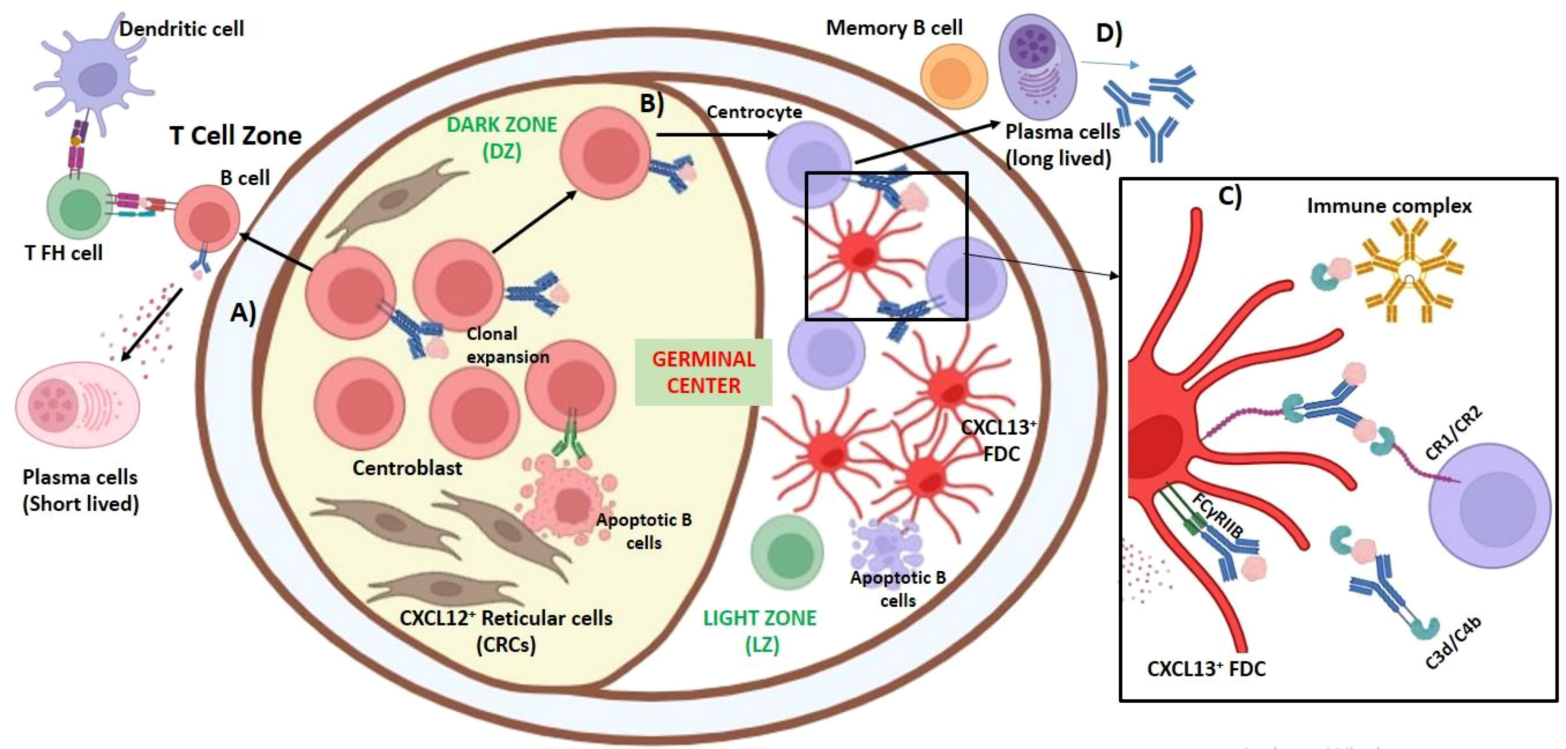
Role of fibroblasts in the development of the germinal center. Germinal centers (GCs) are specialized microstructures that are formed in secondary lymphoid organs (SLOs) during infection or when challenged with T cell-dependent antigens. GC provides a dynamic microenvironment favoring proliferation and differentiation of antigen-activated B cells, leading to the generation of high-affinity plasma cells and memory B cells. Follicular dendritic cells (FDCs) secrete CXCL13 to attract the B cells toward the GC to accommodate these B cells in their extensive processes. Antigen-specific antibody production is governed by the interaction of three cell types: B cells, T cells, and FDCs. **(A)** Once the cognate antigen is recognized by the B-cell receptor, the antigenic peptides are presented to T follicular helper cells (T_FH_); this takes place at the border of T-cell and B-cell follicle zones. This interaction is important for further B-cell activation. B cells migrate to the center of the follicle and undergo clonal expansion, which are known as centroblasts. Some of the blasts migrate to the medullary cords (in lymph node) or toward the red pulp and T zone in between the extrafollicular area (in spleen) and differentiate into plasma cells, which synthesize low-affinity antibodies. **(B)** Centroblasts predominate in the dark zone (DZ), where a dense network of CXCL12-expressing reticular cells (CRCs) is present that promotes homing of B cells during GC response. Somatic hypermutation of IgV_H_ and immunoglobulin class switching from IgM to other immunoglobulins take place during the proliferation of GC B cells. B cells in the GC undergo apoptosis unless they are positively selected. B cells expressing CXCR5, which are now known as centrocytes, migrate to light zone (LZ), which is predominated by CXCL13^+^FCDs**. (C)** At the LZ, FDCs, marked remodeling during the development of germinal centers, internalize immune complexes containing the antigens acquired from non-cognate B cells and recycle them to their surface in their native form for long-term display. Complement receptors CR1 (CD35), which can bind with C3d and CR2 (CD21) with C4b, and Fc receptor FcγRIIB, which binds with IgG, were expressed on FDCs and were found crucial in trapping the immune complexes. The antigen–antibody–C3 complexes formed on FDCs are necessary for the development of B-cell memory. **(D)** These high-affinity centrocytes differentiate into memory B cells and long-lived plasma cells and exist in the GC.

GCs produce memory B cells in preparation for reinfection. Once the cognate antigen is recognized by the B-cell receptor, the antigenic peptides are presented to CD4^+^ T helper cells, and this takes place at the border of the T-cell and B-cell follicle zones. B cells migrate to the center of the follicle and undergo clonal expansion (10, 48). This phenomenon demarcates the GC into two distinct compartments: dark zone (DZ) and light zone (LZ). A dense network of reticular cells expressing CXCL12 (CRCs) can be identified in the DZ (49), which promotes the homing of B cells during GC response and is rich in proliferating cells. Once the somatic hypermutation is complete in the DZ, CXCR4 (a ligand of CXCL12) is downregulated. B cells that express CXCR5 and migrate to LZ are known as centrocytes. The LZ is dominated by FDCs, which are specialized in internalizing and presenting the antigens and undergoing marked remodeling during the development of GCs. The FDCs secrete CXCL13 to attract and present antigens to B cells (51–53). B cells present the antigens retrieved from FDCs to the T cells, CD40/CD40L, and B- and T-cell interactions lead to the positive selection of LZ B cells (52, 53). Studies have suggested that these LZ B cells are Myc^+^ plasma cell precursors (54–58). CXCL13 helps the accumulation of B cells inside follicles and aids in the formation of GCs. The location of CXCL13 is more central than peripheral in the GC (59). In a study where diphtheria toxin (DTx) was injected in mice to induce short-term FDC, the follicular integrity was disorganized in both lymph node and spleen. T lymphocytes and DCs were found localized toward the boundary of the B-cell zone along with the CCL21, which functionally should be restricted in the T-zone area. Thus, in the absence of FDCs, the B-cell zone overlaps with the intermixing region of the CCL21-expressing T lymphocytes and DCs, causing severe loss of GC (60) (**Figure 3**). Apoptosis of B cells in the GCs happens to eliminate self-reactive B cells, which express high levels of ELL (eleven-nineteen lysine-rich leukemia)-associated factor 2 (*Eaf2*). EAF2 is the apoptosis inducer specific for GC B cells (61).

FDCs were earlier considered phagocytic cells, but later, it appeared that the antigens were not internalized but rather trapped in the filiform dendritic processes of the cell, forming a web-like pattern (62–64). Hence, FDCs are non-phagocytic reticular cells that do not express MHC class-II (HLA-DR) molecules, and they have the unique capacity to retain the native antigens in an intact form in the B-cell follicles for a long period (50, 65–67). FDCs endocytose immune complexes containing the antigens acquired from non-cognate B cells and recycle them to their surface in their native form for long-term display (68). This provides an opportunity for the B cells to encounter the antigen for an extended period, FcγRIIB, which binds with IgG expressed on FDC is crucial for trapping the immune complexes (50) (**Figure 3**). FDCs also use complement receptors for trapping antigens; this is explained. Electron microscopy revealed that in some FDCs, the dendritic processes had a chain of interconnected immune complex bodies, termed iccosomes (0.3- to 0.7-μm diameter). Iccosomes derived from FDCs act as a novel mechanism for the delivery of antigens to B cells (69). Iccosomes form a lamellar labyrinth-like structure varying between filiform and glomerulated. In the GCs, these iccosomal antigens are endocytosed by B cells (B220^+^), processed, and presented to T cells, leading to their activation and proliferation (70). Cognate B cells can also acquire FDC-bound immune complexes by capturing CD157 present in FDC or through surface Igs (71).

The complement system is a crucial part of the innate immune system as a first line of defense against infections. The activation of the complement system occurs through three different pathways: classical, lectin, and alternative pathways. These pathways converge at the formation of C3 convertase that culminates in inflammation, opsonization, or cell lysis, through the release of C3a and C5a, the generation of C3b, and the formation of membrane attack complex (MAC), respectively. Excessive activation and tissue damage may be prevented by strict regulation of complement activation by complement receptors that facilitate immune cell interactions and pathogen clearance. Fibroblasts have a significant role in complement regulation and expression of complement receptors (CRs). CRs like CR1 (CD35), CR2 (CD21), CR3, and CR4 (CD11b/CD18) mediate complement-driven cellular responses and contribute to the diverse functions of fibroblasts in immune regulation and tissue homeostasis (72, 73). Excessive complement activation on fibroblasts is implicated in fibrotic diseases (74).

Complement proteins play an indispensable role in the antigen presentation by FDCs. In the GCs of the spleen, aggregates of human IgG are found to be C3-dependent (50, 75). Adult thymectomized mice, when treated with cobra snake venom to deplete C3 level in serum following immunization with dinitrophenylated keyhole limpet hemocyanin (DNP-KLH), exhibited C3 depletion, which led to the impairment of B-cell memory, affecting the precursor proliferation in the splenic lymphoid follicles. The localization of the antigen (DNP-KLH) was also found to be C3 and antibody-dependent (76). Thus, antigen–antibody–C3 complexes formed on FDCs are necessary for the development of B-cell memory; however, the depletion of C3 had minimal effect on the functioning of the already primed B cells. Complement receptors CR1 (CD35), which can bind C3d, CR2 (CD21) with C4b, and Fc receptor FcγR, which binds IgG, are expressed on FDCs and are crucial for trapping the immune complexes (ICs) (50, 77–81) (**Figure 3**). In addition, the expression of CR1 and CR2 on FDC is one of the prerequisites for a strong IgG response (82). FDCs express FcγRIIB at high levels in the GCs but not in the primary follicles. In the lymph node and spleen of FcγRII^−/−^ mice, FDCs exhibit reduced antigen trapping. In a cell culture model when immune complexes were added to antigen-specific T and B cells, antibody response was elevated only when co-cultured with FDCs. Similarly, lymph node lymphocytes from ovalbumin (OVA)-immunized mice were injected into FcγRIIB^−/−^ and irradiated wild-type mice and then after 24 hours injected with anti-OVA immune complexes (preformed ICs). The serum collected after 2 weeks showed robust anti-OVA IgG in the wild type, confirming that the binding of ICs to FcγRIIB makes them highly immunogenic to stimulate B cells. Therefore, the expression of FcγRIIB on FDCs in the GCs acts as a positive regulator of B-cell activation (83).

Efficient capture of ICs by FDCs likely limits the dissemination of pathogens and provides an opportunity for the non-cognate B cells to leave the lymph node without the IC cargo (50, 84). The MZ of the white pulp provides the first line of defense in the spleen. The IgM-bound ICs first localize themselves in the MZ and then associate with MZ B cells with the help of CR1/CR2 receptors. These marginal B cells at the marginal zone between white and red pulp present these IgM-ICs to FDCs in the follicle (85). FDCs release chemoattractant CXCL13 to recruit B lymphocytes and engage them with its CXCR5 receptors; this shuttling is regulated by the balance between CXCR5 and sphingosine-1-phosphate (S1P) receptor, S1P_1_ (86). The localization of IgM-IC is affected in *Cr2* ^−/−^ and C3-depleted mice, suggesting that IgM-IC binding to MZ B cells and antigen deposition on FDCs are complement-dependent (87). To understand the molecular signals specific to FDCs, transgenic mice expressing Cre recombinase controlled by CD21 locus were used. This CD21-cre transgenic mice, where the *cre* gene was inserted at the CD21/35 locus on a bacterial artificial chromosome, showed Cre expression independent of an inducer in mature B cells (88). Thus, the development of the FDC network and B-cell follicle formation are dependent on the expression of p55TNFR (TNF receptor p55) on FDCs that restore VCAM-1, ICAM-1, and CXCL-13 expression in FDCs. However, the effective antibody-mediated immune response is critically dependent on the FDC-specific IKK2 (IκB kinase 2) expression (89). DCs present within the B-cell follicle are capable of sampling FDC-retained antigens before they migrate to the T-cell zone (90, 91). Dysfunction in the FDCs to retain ICs may also limit the peripheral deletion of autoreactive T cells. An increase in the number of autoreactive T cells and their subsequent activation can lead to the loss of B-cell tolerance (92).

## 4 Immunomodulatory role of fibroblasts during infection

Infection or antigenic challenge leads to a huge influx of immune cells into the lymph node parenchyma. The lymphatic vessel increases in size to accommodate more lymphatic fluid, which further activates FRCs to overexpress CCL21 to attract migratory DCs (CCR7^+^) and naïve T lymphocytes, leading to an inflammatory response. To make room for the infiltrating cells, the lymph node undergoes remodeling, i.e., loosening of its reticular network. DCs loaded with antigenic peptides get activated and increase the expression of CLEC-2 (C-type lectin-like receptor-2), which interacts with podoplanin on FRCs (93). This induces the FRCs to lose their contractibility and become elongated, causing the reticular network to relax and the conduit network to expand, enhancing the DC and T-cell mobility and proliferation. IL-1β expression by monocytes and DCs can stimulate the early proliferation of FRCs during antigenic challenge. The inhibition of CCR2 and CCR7 can attenuate the expansion of LNSCs (8). When C57BL/6 mice were challenged with *Escherichia coli* LPS (lipopolysaccharides), and OVA intravenously after 18 hours of T-cell transfer, the expression of several genes was upregulated in FRCs within 12 hours. Among them were the pro-inflammatory chemokines, CCL5 and CXCL9, α-2-macroglobulin, serum amyloid A3 and serine (or cysteine) peptidase inhibitor, clade A, and member 1B (Serpina1d). The upregulation of several interferons (IFNs) and TLR-4 inducible/regulatory genes (e.g., LY6 and LCN2) was also observed. In FRCs as well as BECs, genes related to MHC class II antigen presentation were upregulated (11).

Type I IFNs are secreted by a range of cell types to mediate antiviral response. IFN signaling is mediated through the JAK/STAT signaling pathway, which finally upregulates the expression of IFN-stimulated genes (ISGs) that are crucial for effective antiviral immune response. Approximately 104 ISGs were analyzed in 11 FC clusters in the spleen. Among all the FCs in the spleen, four *Ly6c1* ^+^ FC clusters (*Ly6c1^+^/Hmox1^+^C1q^+^*, *Ly6c1^+^/Hmox1^+^C1q^−^*, *Ly6c1^+^/Hmox1^−^Ccl5lo*, and *Ly6c1^+^/Hmox1^−^Ccl5* ^hi^) showed maximum elevated ISGs (29). Protein kinase R or eukaryotic translation initiation factor 2 alpha kinase 2, *Eif2ak2* (which binds to viral nucleic acid and inhibits protein synthesis at the initiation stage of translation); mixed lineage kinase domain-like pseudokinase, *Mlkl* (a terminal mediator of necroptosis that activates inflammasome synthesis); interferon-induced protein 44, *Ifi44* (upregulated in a wide range of viral infections to restrict viral proliferation); and members of the *Oas*, *Gbp*, *Ifit*, and *Ifitm* families [which are antiretroviral host restriction factors (RFs)] as well as various mediators of viral RNA and DNA sensors such as *Dhx58*, *Ddx60* and *Ifih1*, *Irf7*, and *Zbp1* were overexpressed when *Ly6c1* ^+^ FC was infected with murine cytomegalovirus (29, 94–97). Pezoldt et al. also validated that the upregulation of antiviral signatures in *Ly6c1* ^+^ FC present in red pulp stroma was *Stat1*-dependent, reaffirming the crucial antiviral role played by splenic red pulp stroma (29).

During chronic tissue inflammation in viral or bacterial infections, autoimmunity (rheumatoid arthritis, Sjogren’s syndrome, multiple sclerosis, Hashimoto’s thyroiditis, and autoimmune encephalitis), cancer, age-related disorders chronic allograft rejection, and tertiary lymphoid structures (TLS), ectopic lymphoid tissues are formed in non-lymphoid organs as an immunoprotective response (98). These lymphoid aggregate structures comprising B and T lymphocytes and DCs are supported by a complex network of resident fibroblasts aiding in lymphocyte infiltration through high endothelial venules (99). Various mouse and human experiments so far have identified two subsets of stromal fibroblasts, CXCL13^+^ FDC and CCL21^+^ TRC (100). In the lungs, both bacteria (*Staphylococcus aureus* or *Mycobacterium tuberculosis*) and viruses such as IAV can induce TLS development (98, 101). TLS can also be formed in the liver due to the hepatitis C virus and in the stomach due to *Helicobacter pylori* chronic infection (102). These act as sites for antigen presentation and pathogen clearance. IL-22 is considered a regulatory cytokine for TLS organization in the salivary glands of mice that stimulates the production of CXCL12 and CXCL13 in a virus-induced autoantibody production model (103). Lung fibroblasts during IAV infection express CXCL13 and can drive CXCR5-dependent B-cell recruitment to provide a cross-reactive antibody response (101). In the case of kidney injury during aging, fibroblasts in TLSs get activated and differentiate into a distinct phenotype expressing p75NTR, a neural crest marker, and mature into CXCL13-producing FDCs (104). In human tumors, cancer-associated fibroblasts (CAFs) can organize TLS in a CXCL13-dependent manner, activating lymphotoxin-α_1_β_2_-expressing B lymphocytes as well as tumor-infiltrating lymphocytes (105).

## 5 Conclusion

Fibroblast-driven innate immune responses can be immunostimulatory or immunosuppressive, depending on the nature and the location of the insult in different tissues, resulting in the upregulation of trans-differentiated effector fibroblast phenotypes that modulate the expression and functioning of various innate immune components (PRRs, cytokines, chemokines, and complements) to either resolve or facilitate disease persistence. There is recent surge in studies examining fibroblasts as a therapeutic target in infectious and non-infectious diseases. However, it is vital to fully understand the interacting markers and mediators between fibroblast and immune cells in the heterogeneous milieu of various diseases. The fibroblast heterogeneity needs to be corroborated in different physiological and pathological conditions. Different fibroblast populations program and regulate themselves in order to recreate a lymphocyte-permissive tissue condition and facilitate the spatial orientation and distribution of the cellular infiltrates.

## Glossary

ACKR: atypical chemokine receptor
BAFF: B-cell activating factor
BECs: blood endothelial cells
CAF: cancer associated fibroblasts
cLECs: ceiling LECs
DAMPS: damage-associated molecular pattern
DC: dendritic cell
DNCs: double-negative cells
Dpt: dermatopontin
DZ: dark zone
EAF2: ELL-associated factor 2
ECM: extracellular matrix
ELL: eleven-nineteen lysine-rich leukemia
FAP: fibroblast activation protein-alpha
FCs: fibroblast cells
FDCs: follicular dendritic cells
fLECs: floor LECs
FRCs: fibroblastic reticular cells
FSP1: fibroblast-specific protein 1
GCs: germinal centers
gp38: podoplanin
HEVs: high endothelial venules
IC: immune complexes
ILC1: innate lymphoid cells-1
int-PRC: intermediate PRC
Itgα7: integrin alpha 7
LECs: lymphatic endothelial cells
LNSCs: lymph node stromal cells
LTi: lymphoid tissue inducer
LTo: lymphoid tissue organizer
LT: lymphotoxin
LTβR: lymphotoxin-β receptor
Lyve-1: lymphatic vessel endothelial hyaluronan receptor 1
LZ: light zone
TLS: tertiary lymphoid structures
PRC: perivascular reticular cell
